# Symmetry of learning rate in synaptic plasticity modulates formation of flexible and stable memories

**DOI:** 10.1038/s41598-017-05929-2

**Published:** 2017-07-18

**Authors:** Youngjin Park, Woochul Choi, Se-Bum Paik

**Affiliations:** 1 Department of Bio and Brain Engineering, Korea Advanced Institute of Science and Technology, Daejeon, 34141 Republic of Korea; 2 Program of Brain and Cognitive Engineering, Korea Advanced Institute of Science and Technology, Daejeon, 34141 Republic of Korea

## Abstract

Spike-timing-dependent plasticity (STDP) is considered critical to learning and memory functions in the human brain. Across various types of synapse, STDP is observed as different profiles of Hebbian and anti-Hebbian learning rules. However, the specific roles of diverse STDP profiles in memory formation still remain elusive. Here, we show that the symmetry of the learning rate profile in STDP is crucial to determining the character of stored memory. Using computer simulations, we found that an asymmetric learning rate generates flexible memory that is volatile and easily overwritten by newly appended information. Moreover, a symmetric learning rate generates stable memory that can coexist with newly appended information. In addition, by combining these two conditions, we could realize a hybrid memory type that operates in a way intermediate between stable and flexible memory. Our results demonstrate that various attributes of memory functions may originate from differences in the synaptic stability.

## Introduction

Since Hebb established the concept of activity-dependent synaptic modulation^1^, synaptic plasticity of neural circuits has been considered the key mechanism of learning and memory function. Based on this dogma, there has been a number of efforts to explain the fundamental mechanism of memory—from molecular scale synaptic changes^2^ to population scale memory traces in neural circuits^3^. Extensive research has been done to examine the process of memory allocation^4–6^, how existing memories can be manipulated^7–9^, and how synaptic changes can form a specific type of memory^10^. A recent study also provided evidence of the link between synaptic changes and memory formation^11^. Despite such progress and findings, one important question still remains to be answered: What is the specific mechanism by which neural plasticity forms different types of memory?

Memory can be categorized into two types: flexible and stable forms of memory. The common ground in the dichotomous classifications is that the former decays quickly and is easily replaced by new input, whereas the latter decays relatively slowly and is robust against newly appended input^12, 13^. An intriguing finding from a recent study of the primate caudate nucleus, is that flexible and stable memories can be encoded in the same system^14, 15^. This finding raises the argument that flexible and stable memory might be formed in the same neural circuit, and may not require a completely different mechanism of memory formation. Although a number of theoretical models have been proposed for simulating memories with either flexible or stable features, little is known how to realize both types of memory in the same circuit.

Spike-timing-dependent-plasticity (STDP) is considered a key mechanism for memory formation in neural networks^16–18^ in which synaptic strength is updated according to the exact timing of pre- and postsynaptic spikes^19–21^. Diverse profiles of Hebbian and anti-Hebbian STDP have been observed across various types of post-synaptic cells^22, 23^ and synapses^24–28^. It has been reported that they can also change dynamically depending on the pre- and postsynaptic activity pattern^29^, synaptic cooperativity^30^, and synaptic strength at the moment^19, 20^. Computational models using STDP have successfully reproduced certain features of neuronal memory and memory circuits such as the assembly of inter-neural connectivity, pattern of sequential neural firing and noise robustness^31–33^. However, the mechanism for achieving flexible and stable memory characteristics has not been successfully explained based on these STDP rules.

Here, we introduce a novel model that asserts that the mathematical ‘stability’ of the synaptic learning rule would determine the characteristic of a memory stored in a neural network. Our hypothesis is that alteration of the learning rate symmetry in STDP can differentially regulate the synaptic stability so that it leads to generation of either flexible or stable memories. To test our idea, we designed a computer simulation using a model feedforward network to implement two types of symmetry profiles for learning rate: asymmetric learning rate STDP (AR)^34, 35^ and symmetric learning rate STDP (SR) as examples of two different profiles of synaptic stability. In this model, we defined “memory” as the ability of a system to retrieve consistent response spike patterns when a pre-trained input pattern was received repeatedly. Then we examined the performance of the system in terms of memory “sustainability”—how long the stored memories could last—and “appendability”—how robustly the previously stored memory could survive when new information was appended to memory. Our results showed that the difference in the synaptic stability profile could determine the characteristics of stored memory. Ultimately, we were able to implement a hybrid type of memory from the precise control of learning rate symmetry, which exhibited intermediate properties between flexible and stable memories. Our results may provide new insight for the role of STDP learning rate profile in memory formation.

## Results

### Symmetric and asymmetric learning rate models of STDP

**Figure 1:**
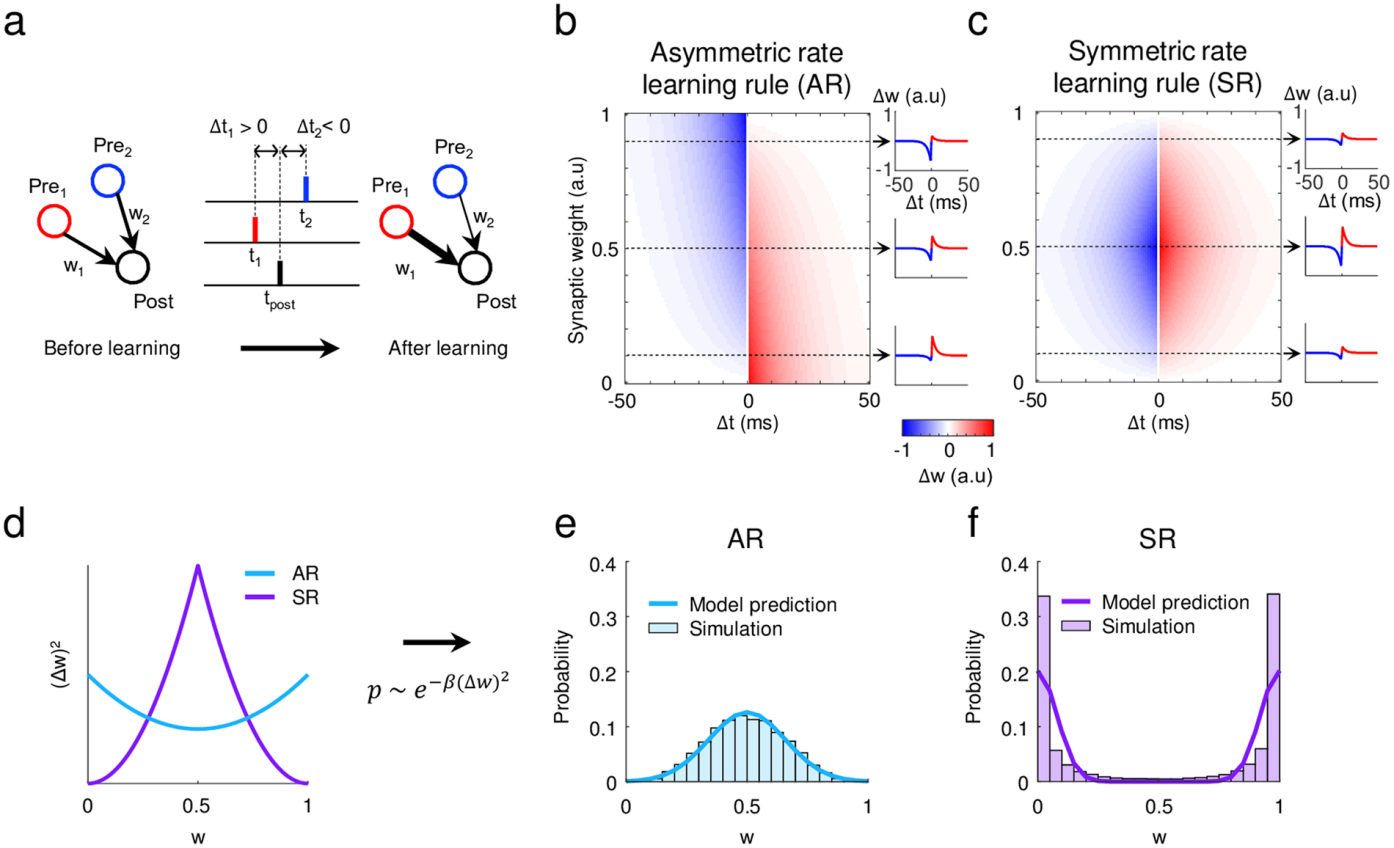
Weight-dependent learning rules: (**a**) A model of spike-timing-dependent plasticity (STDP). Synaptic strength change (Δw) depends on the relative spike timing between pre- and postsynaptic neurons (Δt). Spikes of Δt > 0 leads to long term potentiation (LTP), while spike of Δt < 0 leads to long-term depression (LTD). (**b**) Asymmetric learning rate (AR) model: Learning rates for LTP (red) and LTD (blue) are asymmetric for strong and weak synapses. (**c**) Symmetric learning rate (SR) model: Learning rates for LTP (red) and LTD (blue) are symmetric for strong and weak synapses. (**d**) Instability of the synapses (Δw^2^) for AR and SR models. (**e**,**f**) Weight density function for AR and SR was predicted from Boltzmann distribution (colored lines), and simulated using a single synapse model (histograms).

In a spike-timing-dependent-plasticity (STDP) model, change of synaptic strength or weight (w) depends on relative timing of pre- and postsynaptic spikes, Δt = t_post_ − t_pre_. For example, spikes of Δt > 0 leads to long term potentiation (LTP), while spikes of Δt < 0 leads to long-term depression (LTD) (Fig. 1a). For properly scaled synaptic modulation, the learning rate Δw is considered a function of synaptic strength w (Fig. 1b); so that any particular synapses could not become excessively strong^16, 19, 20, 34^. Here, we consider two models of learning rules—namely, asymmetric and symmetric rate STDP (Fig. 1b,c) where the weight-dependent scaling of learning rate for LTP and LTD is asymmetric or symmetric, respectively. First, asymmetric rate STDP (Fig. 1b) is a typically accepted form of learning rate model, and is sometimes called multiplicative weight dependence in theoretical studies^34, 35^. In this model, the learning rate Δw is biased towards positive (LTP) for weak synapses (w ≈ 0) and is biased towards negative (LTD) for strong synapse (w ≈ 1), so that weak synapses are likely to strengthen while strong synapse are easily weakened. On the other hand, in the symmetric rate STDP model (Fig. 1c), Δw is maximum around the mid-range of w (≈0.5) for both LTP and LTD, and decreases as w increases to ‘1’ or decreases to ‘0’. In this instance, any synapses, once they become very weak or strong by learning, tend to retain their synaptic weights.

To predict the characteristics of each learning rule, we defined the ‘instability’ of the synapses as follows.

1 $$Synaptic\,instability \sim {\sum ({\rm{\Delta }}w)}^{2},$$

Synaptic instability indicates how much the synapse changes from the weight dependent learning rule, thus it is a function of the current value of synaptic weight. From the equations of AR and SR learning rate models in Fig. 1b,c, we mathematically calculated the synaptic instability (Fig. 1d). Then, in order to calculate the probability distribution of the synaptic state, we approximated the density function of w using the Boltzmann distribution of ∆w, by assuming that change of w can be approximated as a binary random-walk process with noisy inputs as

2 $$p \sim {e}^{-\beta {({\rm{\Delta }}w)}^{2}},$$

The estimated model probability distribution predicted that each synapse in the SR model converges to ‘0’ or ‘1’, while in the AR model it converges to 0.5. (Fig. 1e,f, colored lines). We verified this theoretical prediction with a model simulation of a single synapse (see Methods for details). In the simulation, an input neuron was connected to an output neuron and the two neurons were driven by 10 Hz random Poisson spike trains. Then spike pairs were generated randomly to input and output neurons for LTP and LTD, and the synaptic weight was updated by a given STDP rule. As a result, the simulated probability density function well matched the theoretical predictions (Fig. 1e,f, histograms). To summarize, each synapse in the SR model is stable at ‘0’ or ‘1’, while in the AR model, it is stable at 0.5. Due to this difference in instability profile in the learning rule, the SR model will try to retain synaptic weights located at ‘0’ and ‘1’, thus preserving memories well. On the other hand, synapses in the AR model will try to converge at 0.5, thus tending to erase stored memory.

### Both symmetric and asymmetric learning rate models can learn input spike patterns

**Figure 2:**
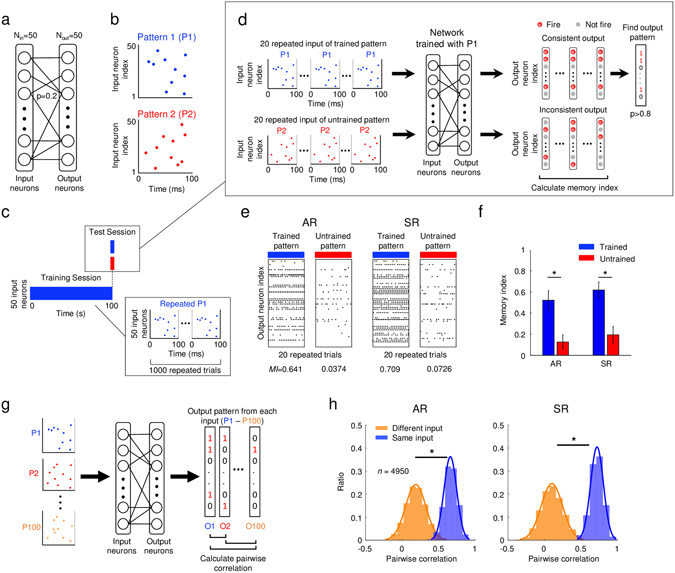
Asymmetric learning rule (AR) and symmetric learning rule (SR) models for volatile/non-volatile memory: (**a**) A feedforward network model for memory: Input neurons (N_in_ = 50) were sparsely connected to output neurons (N_out_ = 50) with connection probability of 0.2. (**b**) Model input spike patterns: Each input neuron generates a spike at random timing within a 100-ms window. (**c**) Memory training and test scheme: In training sessions, a particular input pattern (P1) is fed into a network 1,000 times, for 100 s. (**d**) In each test session, the consistency of network response for trained and untrained patterns were estimated for 20 repeated inputs of identical patterns. (**e**) Response consistency of AR and SR models after training for one input pattern: Both AR and SR systems generated consistent firing patterns for trained input patterns, and inconsistent patterns for untrained input patterns. (**f**) Memory performance of the AR and SR models for 100 input patterns: Both AR and SR networks showed significantly higher memory indices for trained input patterns than for untrained input patterns (Mann-Whitney U-test: *p < 10^−16^, n = 100). Error bars represent the standard deviation. (**g**) To examine how the trained output pattern varies depending on the input pattern, 100 identical networks were trained from 100 different input patterns. The output pattern for each input was measured, and then pairwise correlations between them were calculated. (**h**) Distribution of the pairwise correlation for different and same inputs. The response correlation for different inputs (orange) was significantly lower than that for the same inputs (blue) in both AR and SR cases (Mann-Whitney U-test: *p < 10^−16^, n = 4950).

Using MATLAB, we constructed a model neural network where both input and output layers were composed of 50 leaky integrate-and-fire model neurons (Fig. 2a). Feedforward connections between the two layers were modelled as sparse-and-random synaptic connections, with connection probability of 0.2 between all pairs of input and output neurons. Next, as a simulation of arbitrary information given to the system, we designed “input spike patterns” consisted of 50 input neurons with a 100 ms time window. Every 50 input neurons fired exactly once with random timing within 100 ms such that the firing rate of each neuron was 10 Hz (Fig. 2b). Throughout the study, we used this temporal spike pattern to train our model network. As a simulation of learning and memory processes, we generated the same input patterns 1000 times (100 s in total) to train the network (Fig. 2c). The synaptic weight of each connection was updated using the STDP rules of either asymmetric or symmetric learning rate. For consistency between asymmetric and symmetric learning rate model simulations, all the parameters including the structure of random initial connectivity, synaptic weights, and input patterns were kept identical so that the difference in output activity was induced only from different learning rate profiles (Fig. 1b,c). After the pattern training process, in both the AR and SR models, we observed that the synaptic weights had bimodal distribution, similar to other traditional STDP learning systems^16, 35^ (Supplementary Fig. 1).

After training session of each network, we tested to see if the trained pattern was memorized so that the network could selectively respond to the pattern (Fig. 2d). We observed that both asymmetric and symmetric learning rate model networks could induce a consistent output spike pattern for the trained input; thus could distinguish the trained and untrained input patterns (Fig. 2e). To evaluate quantitatively how consistently the network responded to each trained pattern, we measured the “memory index” as an averaged pairwise cross-correlation between binary patterns of output firings for repeated trials (see Methods for details). As a result, for both asymmetric and symmetric learning rate models, the memory indices for trained pattern were significantly higher than those of untrained patterns, or randomly-ordered series of patterns (Fig. 2f and Supplementary Fig. 2) (Mann-Whitney U-test, p < 10^−16^ for both AR and SR models). We repeated the same simulation with different initial weights and confirmed that the trained and untrained patterns were consistently distinguishable regardless of the initial conditions (Supplementary Fig. 3). This result indicates that both learning rate models are capable of training networks to memorize and identify temporal spike patterns.

Then, to investigate how the trained output pattern varies depending on the input pattern^36^, we examined variation in the response pattern of the output neurons, which indicates a set of neurons consistently respond (above 80% chance) to a trained input (Fig. 2d, output pattern). We extracted 100 “output patterns” from 100 different input patterns, and calculated the pairwise correlations within 100 output patterns (Fig. 2g). Then, the distribution of pairwise correlation across different inputs was compared to that within the same input. In both the AR and SR models, we found that the response correlation for different inputs (Fig. 2h, orange) was significantly lower than that for same inputs (Fig. 2h, blue) (Mann-Whitney U-test, p < 10^−16^ for both AR and SR models). Therefore, we concluded that output patterns after training for different inputs are readily distinguishable in our model, whereas output patterns after training for the same inputs are fairly consistent.

### Symmetric learning rate model can form stable memory

**Figure 3:**
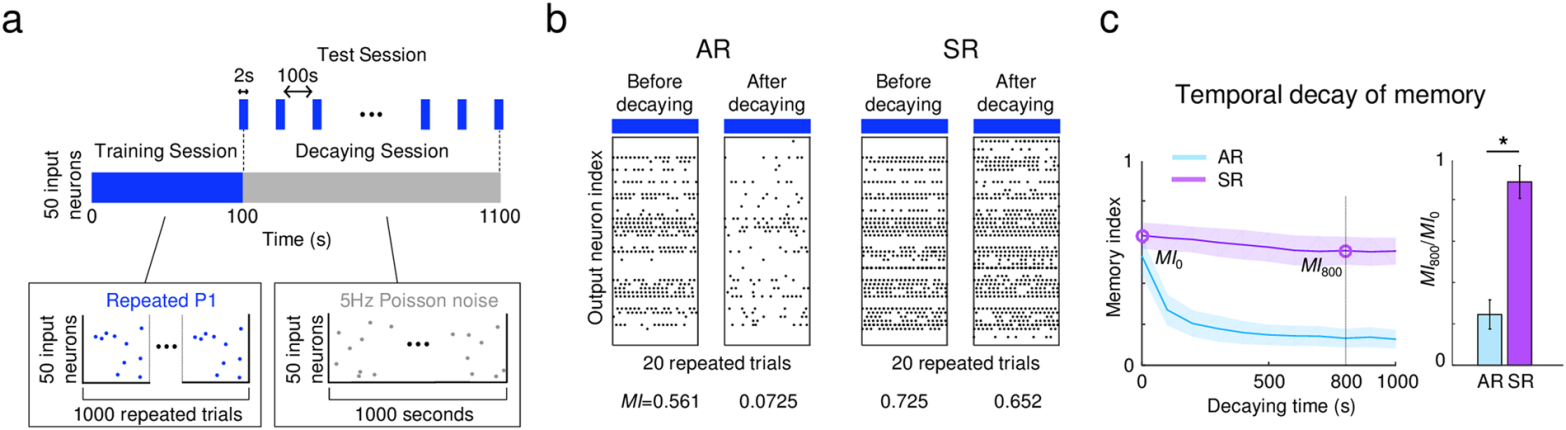
Comparison of AR and SR models for decaying by noise (**a**) Test scheme of memory decay: After 100 s of training, 5-Hz Poisson spikes were introduced to the networks for 1000 s to erase the stored memory. In decay sessions, we estimated the memory index every 100 s. (**b**) Response of AR and SR models after 1000 s of decay: The response pattern of the AR model became inconsistent output firing after the decay session, while the SR model showed consistent output firing even after decaying. (**c**) The time course of the memory index during the decay session: Memories in the AR model decayed rapidly, while those of the SR model did not show noticeable decay. The memory index ratio (*MI*_800_/*MI*_0_) of SR was significantly higher than that of AR (Mann-Whitney U-test: *p < 10^−16^, n = 100). Shaded area and error bars represent the standard deviation.

The amount of memory decaying over time is a distinct factor in determining the degree of memory stability^12^. To test the decaying characteristics of each model after training with a particular input pattern, we introduced a 5-Hz Poisson noise input to the network as simulation of spontaneous neural activity in the network (Fig. 3a, decaying session). During the decaying session, we measured the memory index for every 100 s, to estimate the temporal degradation of memory (Fig. 3a, test session). As a result, we observed that the responses of the AR model after the decaying session were noticeably disturbed, indicating that the stored memory pattern was being erased, while that of the SR model remained nearly intact (Fig. 3b). Thus, the estimated memory index of the AR model decreased significantly whereas the SR model did not show a noticeable drop in the index.

To quantify how much the initial memory was being preserved at each moment during the decaying session, we investigated the time-course of the memory index change (Fig. 3c). As expected, the memory decay over time appeared significant only in the AR model where the initial value of memory index quickly dropped as soon as the noise was introduced. Then we calculated the ratio of indices before the decaying session (*MI*_0_) and after 800 s of decaying (*MI*_800_). The ratio of the SR model, *MI*_800_/*MI*_0_ = 0.8848 was significantly higher than that of the AR model, 0.2452 (Mann-Whitney U-test, p < 10^−16^). We repeated the same simulation under different conditions of noise level, and confirmed that the observed difference between the AR and SR models was fairly consistent (Supplementary Fig. 4). To investigate how the SR model preserves old memories at the synaptic level, we examined synaptic weight changes during the decay process (Supplementary Fig. 5). During the decaying session, a bimodal distribution of the synaptic weights was sustained in the SR but not in the AR model. Given that synaptic weights hold memory information in this system, sustained bimodal weights in the SR model would be more likely to preserve old memory. Overall, these results indicate that the SR model can encode non-volatile or stable memory, while the AR model encodes more volatile or flexible memories.

### Symmetric learning rate model can append new memories

**Figure 4:**
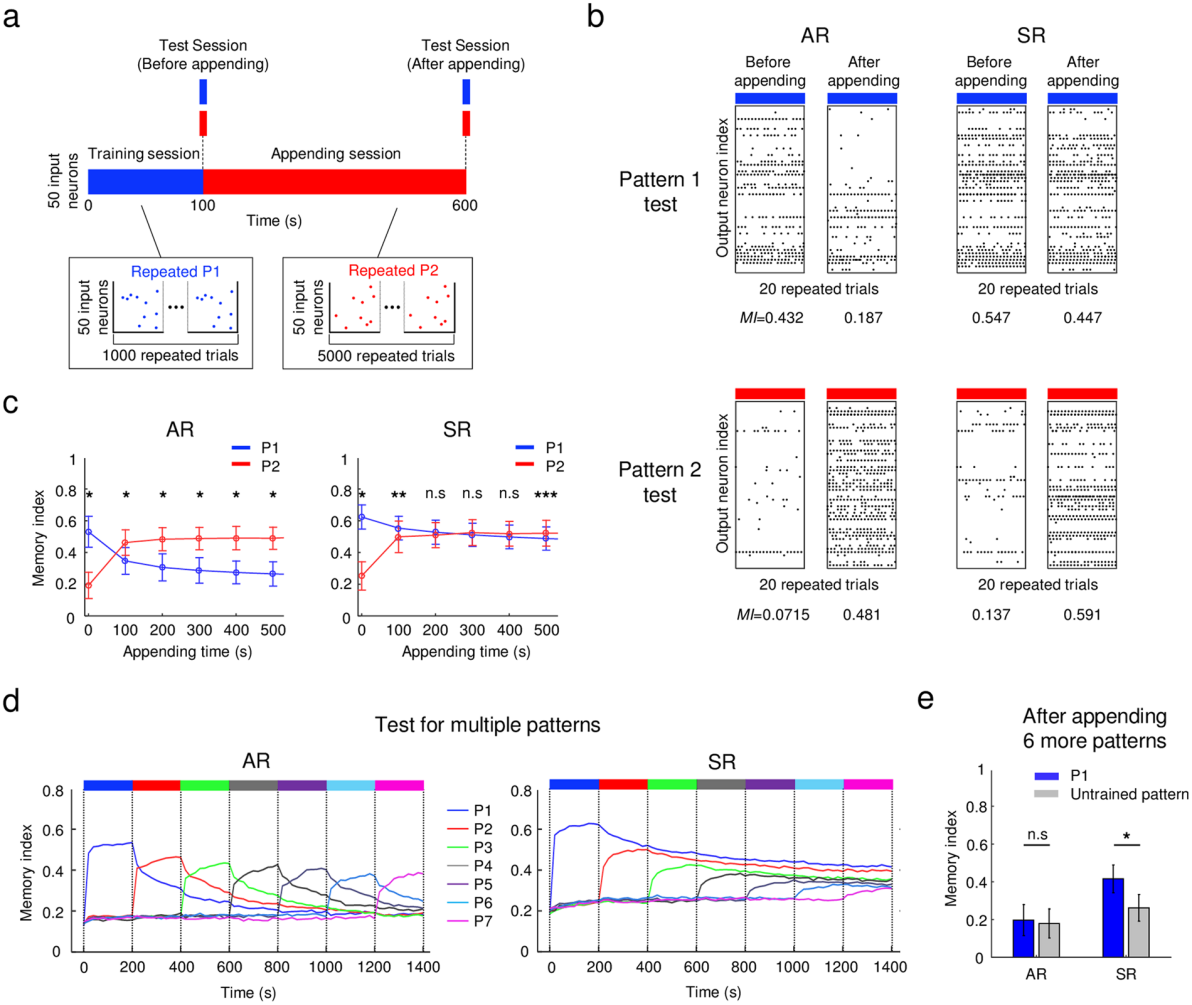
Comparison of AR and SR models for appending new memories: (**a**) Test scheme for appending memory: After 100 s of training with pattern P1, another input pattern P2 was trained for 500 s to test the ability of each model to append memory, and the memory indices for P1 and P2 were measured before and after each appending session. (**b**) The response of each model to old and new input patterns: The AR model lost P1 memory after appending P2 (left), while the SR model did not lose P1 memory even after appending new memory (right). (**c**) Memory index change during appending session: In the AR model, the memory index for the old pattern (P1) decayed fast as the memory of the new pattern (P2) formed. In the SR model, the memory index for P1 slightly decreased but was kept as high as that of the newly formed memory of P2 (Mann-Whitney U-test: *p < 10^−16^, **p < 10^−4^, ***p < 0.01, n = 100). (**d**) Memory index change for appending multiple patterns: Different temporal patterns (P1-P7) were sequentially introduced to the model networks: In the AR model, old memories rapidly decayed as soon as new patterns were introduced. In the SR model, both old memories and new memories were kept together, well above the response level for the untrained inputs. (**e**) P1 memory index after appending six new patterns. In the AR model, the memory index of P1 was remarkably decreased, indistinguishable from that of an untrained pattern (Mann-Whitney U-test, p = 0.2232, n = 100). On the other hand, in the SR model, the memory index of the P1 after training was still significantly higher than that of an untrained input (Mann-Whitney U-test: *p < 10^−16^, n = 100). Error bars represent the standard deviation.

The ability to collect multiple memories is also a crucial feature of stable memory. For this, newly appended memories must not degrade previously stored memories. To test if this memory accumulation can be achieved in our models, both AR and SR networks were trained with multiple input patterns provided consecutively (Fig. 4a). First, we trained the network to memorize a pattern P1 for 100 s (training session), then we retrained the network with another pattern P2 (appending session). We tested the memory performance of the network response to P1 and P2, for every 100 s during the appending session. We first observed that both asymmetric and symmetric rate models showed a consistent response pattern for P2 after appending, confirming that the pattern P2 was memorized (Fig. 4b, bottom). In the AR model, however, the network response to P1 was noticeably altered, indicating that the previously stored memory was disturbed by the newly appended memory (Fig. 4b, top left). On the other hand, in the SR model, the response activity to pattern P1 was observed to be consistent even after appending (Fig. 4b, top right), suggesting that the information of both P1 and P2 was memorized in the circuit. For further quantitative analysis, we measured the instantaneous values of memory index, for every 100 s during the appending session (Fig. 4c). As expected, the memory index for P1 in the AR model decreased rapidly as soon as the training of P2 started, while that in the SR system changed only slightly.

We repeated the same test for multiple inputs. We sequentially gave seven different input spike patterns, each for 200 s, to both the AR and SR models. Then we assessed the memory index of each pattern during training (Fig. 4d). In the AR model, the trace of old patterns rapidly decayed, to be replaced by newly appended memories. The memory index of the pattern P1 after appending six different new patterns (P2-P7) was markedly reduced (Fig. 4d, left and Fig. 4e, left). The condition became indistinguishable from that of an untrained pattern (Mann-Whitney U-test, p = 0.2232). On the other hand, in the SR model, the memory of old pattern was preserved. The memory index of the pattern P1 after training of P2–P7 was reduced to some extent, but still significantly higher than that for untrained input (Mann-Whitney U-test: *p < 10^−16^) (Fig. 4d, right and Fig. 4e, right). Therefore, we confirmed that the memory of P1 in the AR model was lost after training with new patterns, whereas the memory of P1 remained after the accumulation of new memories in the SR system. This also indicates that the memory in the SR model network cannot be easily erased, because it will accumulate all the input patterns that the capacity of the system allows. To investigate how memories are allocated in the synapses of the network during this process, we measured the ratio of synaptic weight, which converges to ‘0’ or ‘1’, and indicated the information contained (Supplementary Fig. 6). As the number of appending patterns increased, in the SR model, the ratio of synaptic weights converged to either ‘0’ or ‘1’ monotonically increased, while the ratio of converged weights of AR model remained constant (≈0.4) through appending session. This result shows that, in the SR model, new inputs were stacked in available synapses that did not contain previous information, while in the AR model, new inputs were overwritten on synapses storing previous memories.

### Linear combination of two learning models generates a hybrid memory

**Figure 5:**
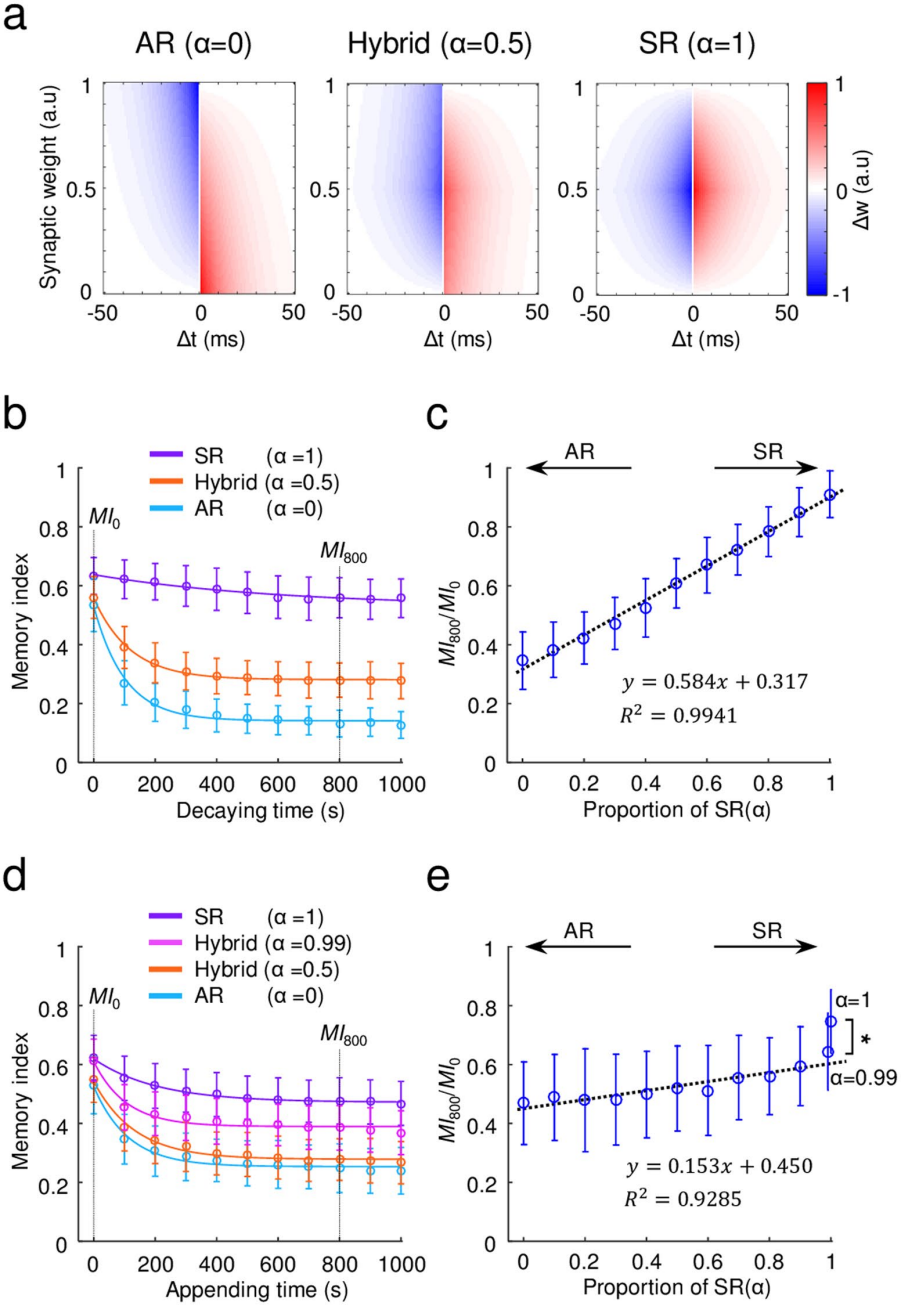
A hybrid memory model: (**a**) Hybrid learning rule was defined as the weighted sum of AR and SR models, and the portion of SR was denoted by α. (**b**) Temporal decay of the memory in the hybrid model was tested as in Fig. 3c. The hybrid model with α = 0.5 showed intermediate characteristics between the AR and SR models (orange line). The error bars represent the standard deviation for 100 trials. (**c**) Modulation of memory decay for various values of α: As α increased, the ratio of memory indices (*MI*_800_/*MI*_0_) increased linearly, suggesting that the characteristics of the hybrid model make a linear transition from the AR to the SR model. (**d**) The decay of old memory with newly appended memory in the hybrid model was tested as in Fig. 4c. The memory index in the hybrid models with α = 0.5 and 0.99 were intermediate values between those of the AR and SR models, but showed nonlinear transition as α varied. (**e**) The proportion of old memory was maintained as α varied: As α increased from 0 to 0.99, the ratio of memory indices (*MI*_800_/*MI*_0_) increased linearly. After this, the ratio abruptly increased as α changed from 0.99 to 1 (Wilcoxon signed rank test: *p < 10^−9^, n = 100). The data point at α = 1 was excluded from the linear regression (dashed line). The error bars represent the standard deviation.

Next, we tested to see if we could create a different type of memory system by combining the AR and SR models. For this, we linearly integrated two STDP kernels, taking the weighted summation of the asymmetric and symmetric learning rate profiles (Fig. 5a), with a parameter α as the proportion of symmetric rate STDP in the linear combination. By regulating α, we could control the properties of new memory, making them closer to AR (α = 0) or SR (α = 1) models. As a result, for 0 < α < 1, we could create a new type of memory that decayed slower than AR but faster than in the SR model, and that had an intermediate feature of memory appendability between that in the AR and SR models. We refer to this type of memory as “hybrid memory.”

First, to examine the decay properties of a hybrid memory (α = 0.5), the network was tested in a decaying session (5 Hz Poisson noise, 1000 s) after training of an input pattern, as previously. Once every 100 s during the decaying session, we measured the memory index. As expected, the hybrid network showed intermediate characteristics in the memory decay test: the stored memory decayed faster than in the SR, but slower than in the AR models (Fig. 5b). In addition, as α increased, the ratio of the memory indices, *MI*_800_/*MI*_0_ increased linearly (Fig. 5c). This suggests that the transition from AR to SR model could be continuous.

Similarly, we also examined the memory appending feature of the hybrid model. With models of various α, we trained the network with a pattern P1 for 100 s, and then trained with another pattern P2 for 1000 s, while estimating the memory indices of pattern P1 for every 100 s. Different from the memory decay feature, the hybrid memory showed a nonlinear transition from AR to SR model. As we increased α from 0 to 1, the profile of the memory index of the seed pattern P1 in the hybrid memory model, differed only slightly from that of the AR model, up to a fairly high value of α (≈0.9) (Fig. 5d). For a very high value of α (near 1), the profile of the memory index suddenly transitioned to that of the SR model (Fig. 5d, α = 0.99). To examine this transition quantitatively, we measured the ratio of memory indices *MI*_800_/*MI*_0_ while varying α. As α increased from 0 to 0.99, *MI*_800_/*MI*_0_ slightly increased, but suddenly spiked when α increased from 0.99 to 1 (Fig. 5e, Wilcoxon signed rank test, p < 10^−9^). This implies that, the memory appending feature may not be consistent with the memory decaying feature in the model. For quantitative analysis on this nonlinear transition for varied α, we fitted each decay curve in Fig. 5b,d to an exponential function and examined the fitted parameters (Supplementary Fig. 7). As a result, we found that the decay rate, amplitude, and asymptote of the fitted curves showed a linear change for α < 0.9, and then spiked nonlinearly, near α = 1. Therefore, we concluded that the hybrid model showed a linear transition in its memory feature in a large regime of parameters, and became nonlinear only near α = 1 (like the pure SR model), which showed a stable memory characteristic.

## Discussion

In this study, we introduced our hypothesis that the stability profile of the synaptic learning rule would determine the characteristic of a memory stored in a neural network, and showed that our simulated results supported this idea. The SR model retained encoded memories without being affected by noise or new memories and did not lose its ability to learn new information. On the other hand, the AR model continuously replaced stored memories with newly encoded inputs, and the hybrid model showed intermediate properties between those of the SR and AR models. In the current study, we focused on comparing SR and AR models to illustrate our theoretical idea regarding the effect of the synaptic stability and the STDP profile on memory function. However, these models are two extreme cases of our conceptual model, whereas the most generalized form of our theoretical model is the hybrid case with characteristics of both models. In addition, considering that (1) stability and flexibility are both basic properties of memory circuits, (2) the relative degree of stability and flexibility may vary from circuit to circuit, we may also suggest that our hybrid model is a reasonable design for describing memory characteristics of various circuits. For these reasons, we suggest that the hybrid model is the most realistic form of STDP that we may expect to observe in the experimental data. Various local neural circuits involved in memory function may have different properties in terms of stability and flexibility. This can be readily assessed by our hybrid model, and an experimental study may be able to observe various STDP profiles in local circuits, providing a good match to our hybrid model. In addition, these results may account for the mechanism of selectively controlled memory storage in the brain that, in turn, allows adaptation to both fast-changing and long-lasting environments.

One promising candidate with which to test this idea is the basal ganglia system, where flexible and stable value information is coded distinctively in the caudate nucleus subregions^14, 15^. Previous studies reported that flexible and stable value information are coded distinctively in the caudate nucleus subregions: the caudate “head” responded to flexible values, whereas the caudate “tail” was more sensitive to stable values. More interestingly, the neurons in the caudate “body” responded to both flexible and stable values in a mixed manner. This finding implies that fast-changing and long-lasting memories can potentially be formed in very similar neural circuits, and our model can provide a plausible explanation for the formation of flexible and stable memories within local neural circuits. This experimental observation can be readily reproduced in our model network in which the STDP learning rate symmetry profile gradually varies depending on where a neuron is located in the caudate nucleus. Using a hybrid STDP model, even the intermediate memory property of the caudate “body” can be readily realized.

Our finding that STDP symmetry is a potential key determinant of memory type can be explained in terms of synaptic strength stability. A number of theoretical circuit studies propose that distribution of synaptic strength tends to become bimodal during learning^16, 35, 37, 38^, and that the bimodally converged synapses are thought to play an important role in memory storage. In our model simulations, we confirmed that synapse strengths converged to either minimum or maximum value during the learning process, indicating that information is stored in these converged synapses (Supplementary Figs 1 and 3). In the SR model, the weight of a synapse is bi-stable at its minimum and maximum due to its stability profile; thus, a converged synapse cannot easily escape from its converged weight value (Supplementary Fig. 5). Notably, the network can store new information until every single synaptic connection is converged to minimum or maximum, and thus the number of available synaptic connections in the network will determine the memory capacity of the system. On the other hand, due to the synaptic stability profile of the AR model^34, 35, 39^, converged synapse strength could easily decay toward the mid-range value of weight by noise or other inputs, similar to the decay of stored memory in previous simulation studies on memory lifetime^40, 41^.

It is also notable that, even though we used a spike-pair-based model, our model does not require any particular type of STDP model. Our model idea is simply that memory performance depends on the STDP instability profile, regardless of the specific details of the type of STDP model. For this reason, we chose the spike-pair-based model as the simplest form of STDP, but adequate to generate different profiles of synaptic stability. We confirmed that our main result is consistent even with different types of STDP models such as the voltage-based model (Supplementary Fig. 8), as long as the profile of synaptic stability is designed similarly.

One of the distinctive features of our simulation is that the measured memory performance in terms of consistency of output spike pattern, which is assumed to be the formation of a memory engram, was compatible with previous observations. In fear-conditioning experiments^8, 42^, a consistent formation of neural firing pattern was reported in mice that showed well trained freezing behavior. Thus, in our model, a consistent firing pattern of output neurons approximates the selective firing of neurons or the structure of an engram in the hippocampus or lateral amygdala. In this way, we could quantitatively simulate the process of memory formation, decay and modulation, in terms of firing pattern consistency, and could also estimate the “sustainability” and “appendability” of stored memory.

Although we successfully showed the characteristics of memories formed with different STDP learning rate profiles, a number of additional studies ought to be conducted to confirm the biological validity of the model. Firstly, for simplicity, the model network used in this study does not contain any inhibitory interactions. Probably, lateral inhibition plays a crucial role in competition between neuronal assemblies during memory formation^43–45^. Although we focused on the effect of different STDP learning rate symmetry in the current study, we will extend our work to consider a more complete scenario on the mechanism of dynamic memory formation by including lateral inhibition, neural competition and synchronized neural activities. Secondly, in the simulations, we introduced some amount of noise fluctuation in the neural membrane potential which facilitated spike generation in the initial stage of learning by stochastic resonance. Stochastic resonance allowed initiation of synapse strength modulation by STDP that required output spikes induced by input spikes. Considering the fact that the amplitude of noise used in our simulation was higher than that reported in experiments^46^, noise may not be the only source of initiation of synaptic modulation. For example, another possible candidate is spontaneous gamma oscillation^47–49^, commonly observed across various brain regions. It is known that the amplitude and phase of gamma oscillation can be dynamically modulated by various network properties, such as theta frequency rhythm or local inhibition level. Therefore, we believe that the introduction of spontaneous gamma oscillation to the network may play a role in the initiation of spike-timing-dependent synaptic modulation by stochastic resonance, as well as dynamic control of learning and memory. Realization of this scenario will also be an important part of our follow-up studies. Lastly, in this case we manually balanced the network activity, but doing so was limited to a specific range of input firing rates. In order to make the system stable for any arbitrary input, we believe a homeostatic plasticity mechanism^50^ should be applied to the system in future studies.

Although one of the key components of our model, the symmetric learning rate profile, is yet to be explicitly observed in experiments, our results can still be valid not only with SR and AR models, but with any other plasticity model that could generate a stable and an unstable condition of learning. The bottom line of the SR model is that the synapses become stable as synaptic strength becomes either very strong or very weak. This condition can be achieved by various factors, such as neuromodulator that affects to LTP and LTD simultaneously^51, 52^, gain modulation or controlled excitability^53^ by feedback loop that temporally inhibits spike generation of target neurons with recently strengthened synapses. These scenarios can be validated experimentally, based on our theoretical prediction.

Overall, we propose a simple but powerful model that explains important features of memory formation. We believe that our model can shed light on the study of how memory is formed, erased and controlled.

## Methods

### Single Neuron Model

All simulations were performed using MATLAB codes. For a single neuron simulation, we used a leaky-integrate-and-fire neuron model. The membrane voltage of neuron j at time t can be updated by:

3 $$C\frac{d{V}_{j}(t)}{dt}={g}_{L}({E}_{L}-{V}_{j}(t))+{g}_{j}(t)({E}_{syn}-{V}_{j}(t))+{I}_{noise},$$

where C is membrane capacitance, g_L_ is leak conductance, E_L_ is resting potential, and E_syn_ is reversal potential. We used the commonly accepted values for physiological parameters (C = 1 μF, g_L_ = 0.4 μS, E_L_ = −65 mV, E_syn_ = −5 mV, dt = 1 ms). A Gaussian noise current, I_noise_ is given to each neuron with mean 0 and standard deviation 1.2 nA. Voltage gated channel conductance g_j_ is determined by the following equation

4 $$\frac{d{g}_{j}(t)}{dt}=-\frac{{g}_{j}(t)}{{\tau }_{syn}}+{c}_{syn}\sum _{i\in input}{w}_{ij}{S}_{i}(t),$$

where S_i_(t) denotes the spike train of presynaptic neuron i, and w_ij_ means synaptic weight between pre- and post-synaptic neuron. Time constant τ_syn_ determines the decay speed of EPSP and c_syn_ implicates the size of the excitatory postsynaptic conductance evoked by an input spike. When V_j_ reaches E_threshold_ = 55 mV, an action potential is generated immediately and V_j_ is reset to resting potential E_L_. We used the commonly accepted values for these parameters (τ_syn_ = 3 ms, c_syn_ = 0.12 μS ms^−1^, dt = 1 ms).

### Mathematical Model of Synaptic Plasticity

The update of the synaptic weight is determined by the spike timing interval Δt = t_post_ − t_pre_ as in the following equations:

5 $${\rm{\Delta }}{w}_{ij}=\begin{array}{c}{\varepsilon }_{+}({w}_{ij})\cdot {k}_{+}{e}^{-\frac{{\rm{\Delta }}t}{{\tau }_{+}}}\quad {\rm{\Delta }}t > 0,{\rm{LTP}}\\ {\varepsilon }_{-}({w}_{ij})\cdot {k}_{-}{e}^{-\frac{{\rm{\Delta }}t}{{\tau }_{-}}}\quad {\rm{\Delta }}t\le 0,{\rm{LTD}}\end{array}$$

where k and τ are parameters that determine the amplitude and decay of STDP kernel, and ε_+_(w) and ε_−_(w) denote weight-dependent learning rates for positive and negative values of Δt. We set k_+_ = 0.06, k_−_ = −0.09, τ_+_ = 3 ms, τ_−_ = 15 ms. To make w stay in range w_min_ < w_ij_ < w_max_, we implemented three different learning rate profiles: asymmetric (AR), symmetric (SR), and hybrid. The AR model, frequently called the multiplicative learning rate^34, 35^, is defined as

6 $$\begin{array}{c}{\varepsilon }_{AR+}({w}_{ij})={w}_{{\rm{\max }}}-{w}_{ij}\\ {\varepsilon }_{AR-}({w}_{ij})={w}_{ij}-{w}_{{\rm{\min }}}\end{array}$$

where w_max_ = 1 and w_min_ = 0. In this model, the weight change (Δw) is maximum at w = 0 for LTP and w = 1 for LTD.

On the other hand, the SR model uses a two-sided, linear-bound method as in

7 $${\varepsilon }_{SR+}({w}_{ij})={\varepsilon }_{SR-}({w}_{ij})=2\cdot \,{\rm{\min }}({w}_{{\rm{\max }}}-{w}_{ij},{w}_{ij}-{w}_{{\rm{\min }}})$$

The hybrid model uses a linear combination of ε_AR_ and ε_SR_ as

8 $${\varepsilon }_{HY}({w}_{ij})=\alpha {\varepsilon }_{SR}({w}_{ij})+(1-\alpha ){\varepsilon }_{AR}({w}_{ij}),\quad 0 < \alpha  < 1,$$

where α denotes the proportion of the symmetric rate STDP rule.

The synaptic instability (Fig. 1d) was defined as

9 $$Synaptic\,instability=\sum {({\rm{\Delta }}w)}^{2} \sim {({\varepsilon }_{+})}^{2}+{({\varepsilon }_{-})}^{2},$$

The learning rates of LTP and LTD were estimated separately, due to the asymmetry of the AR model.

The probability density function of w was approximated using the Boltzmann distribution of ∆w, by assuming that change of w can be approximated as a binary random walk process with noisy inputs as

10 $$p \sim {e}^{-\beta {({\rm{\Delta }}w)}^{2}},$$

### Model Simulation of a Single Synapse

We designed a single synapse model that consisted of one presynaptic neuron, one postsynaptic neuron, and their connection (Fig. 1e). The synaptic weight between the pre- and postsynaptic neuron was initialized to a random value between ‘0’ and ‘1’. Two neurons were driven by 10 Hz random Poisson spike trains for 1000 s. Then spike pairs to the input and output neurons for LTP and LTD were generated randomly, and the synaptic weight was updated by the given STDP rule, either AR or SR. We examined the probability distributions in 10,000 trials of how weights changed after sufficient time (1000 s).

### Model Neural Network

In this study, the model network we used consists of two layers, 50 excitatory input neurons, and 50 output neurons, with sparse random feedforward connections. The connection probability between each input and output neuron was set to 0.2. Initially, the synaptic weights or connection strength between input and output neurons were randomly sampled from normal distribution, with mean 0.5 and standard deviation 0.05.

### Model Input Pattern for Memory Training

To train the network, we designed “input spike patterns” consisting of 50 input neurons with a 100 ms time window. Every 50 input neurons fired exactly once with a random timing within 100 ms such that the firing rate of each neuron was 10 Hz. Training for each pattern was done for 100 s by feeding 1000 successive identical patterns with no delay.

### Test of Memory Performance

To confirm the network had “memorized” a trained pattern, we tested the response of the network with a trained and an untrained pattern. Each test input pattern was given repeatedly (20 times), and we represented firings of output neurons as a binary number—if it fires at least once, then ‘1’—to simply indicate if a neuron is involved in the memory pattern. Even if an output neuron fired more than once per trial, we counted it as ‘1’ (fire). When there was no firing at all during one trial, we counted it as ‘0’ (not fired). Then we calculated the memory index from these 20 binary response vectors. The memory index was defined as the average pairwise cross-correlation between output neuronal firing for repeated inputs as

11 $$MI=\frac{1}{{N}_{pair}}\sum _{m,n\in [1:20]}\frac{{S}_{m}\cdot {S}_{n}}{{N}_{firing}},$$

where S_m_ denotes the m^th^ binary vector of output firing, N_pair_ is the number of all possible pairs, _20_C_2_ = 190, and N_firing_ is the number of output neurons fired at least once during 20 repetitions of input. Thus, the memory index is normalized in the range from 0 to 1.

### Test of the Variation of Output Pattern

To examine how the output pattern varies depending on the input pattern, we defined the “output response pattern” as a set of neurons that consistently (above 80% chance) respond to a trained input. Thus, this “output pattern” represents a set of neurons involved in memory after the training. We compared this output pattern within the same input and across different inputs. From “100 different input patterns” and “100 same input patterns”, we trained the 100 identical networks and got 100 output patterns in the form of binary vectors. Then we calculated the pairwise correlations within 100 output patterns (total 4950 pairs). The response correlation “within the same input” and “across different inputs” was compared (Fig. 2h).

### Test of Memory Decay

To simulate the temporal decay of memory with background noise, a 5-Hz Poisson random spike train was given to the trained network for T seconds. To quantify the amount of memory maintained in the network, the ratio between the memory index at t = T and at t = 0 was estimated as

12 $${\rm{Maintained}}\,{\rm{memory}}\,{\rm{ratio}}=\frac{M{I}_{t=T}}{M{I}_{t=0}},$$

where t denotes the time in the decay session.

## Electronic supplementary material

Supplementary Information
