# Supplementary material for: Symmetry of learning rate in synaptic plasticity modulates formation of flexible and stable memories
Source: Sci Rep. 2017 Jul 18;7:5671. doi: 10.1038/s41598-017-05929-2 (PMC5516032; doi:10.1038/s41598-017-05929-2)
Supplement: Supplementary file 1 — Supplementary Information [file 41598_2017_5929_MOESM1_ESM.pdf]

Symmetry of learning rate in synaptic plasticity modulates formation of flexible and stable memories

Youngjin Park[1], Woochul Choi[1,2] & Se-Bum Paik[1,2 *]

[1]Department of Bio and Brain Engineering, [2]Program of Brain and Cognitive Engineering, Korea Advanced Institute of Science and Technology, Daejeon 34141, Republic of Korea

*email: sbpaik@kaist.ac.kr

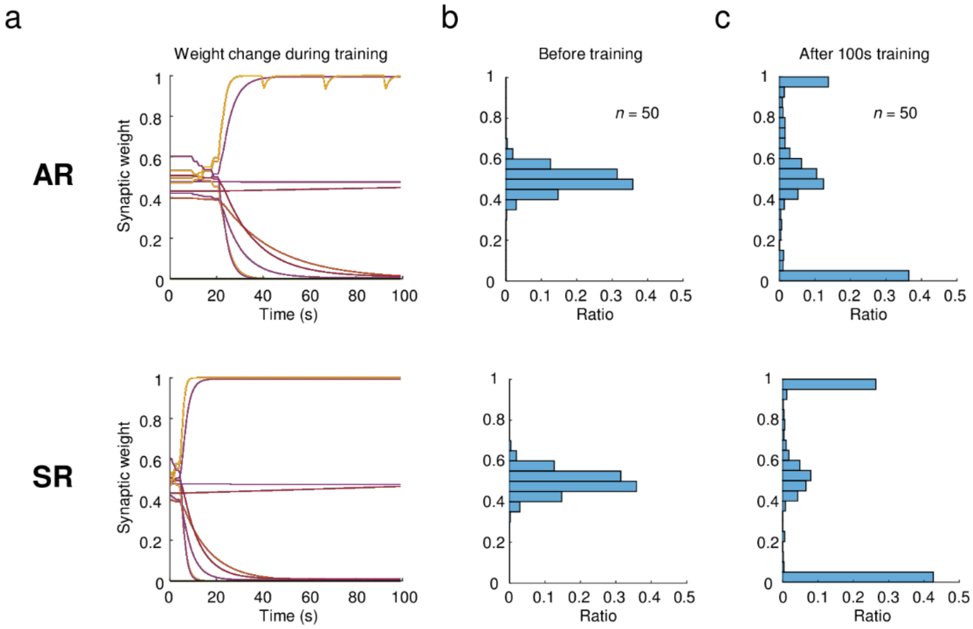

Supplementary Fig. 1. Synaptic weight change during training. (a) An example figure showing the weight changes in synapses connected to one output neuron during the training session. Synapses were converged to 1 by LTP or converged to 0 by LTD. Synapses which were uncorrelated with the output did not change. (b) Synaptic weight distribution before training (mean=0.5, s.d.=0.05). (c) The synaptic weight distributions become bimodal after training in both the AR (upper) and the SR (lower).

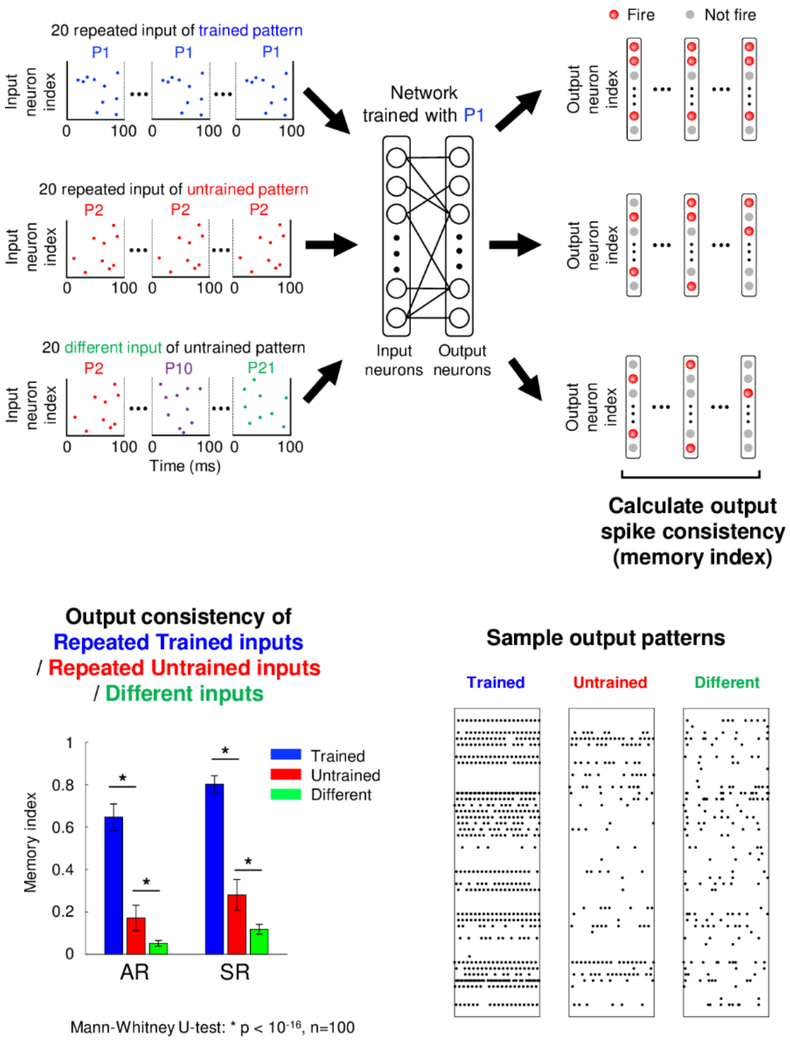

Supplementary Fig. 2. Response variation in each trial under various conditions of input: Repeated input of trained pattern, repeated input of untrained pattern, and a randomly-ordered series of untrained patterns. Repeated trained inputs induced much higher MI than did repeated untrained inputs. A random series of untrained inputs showed the lowest MI.

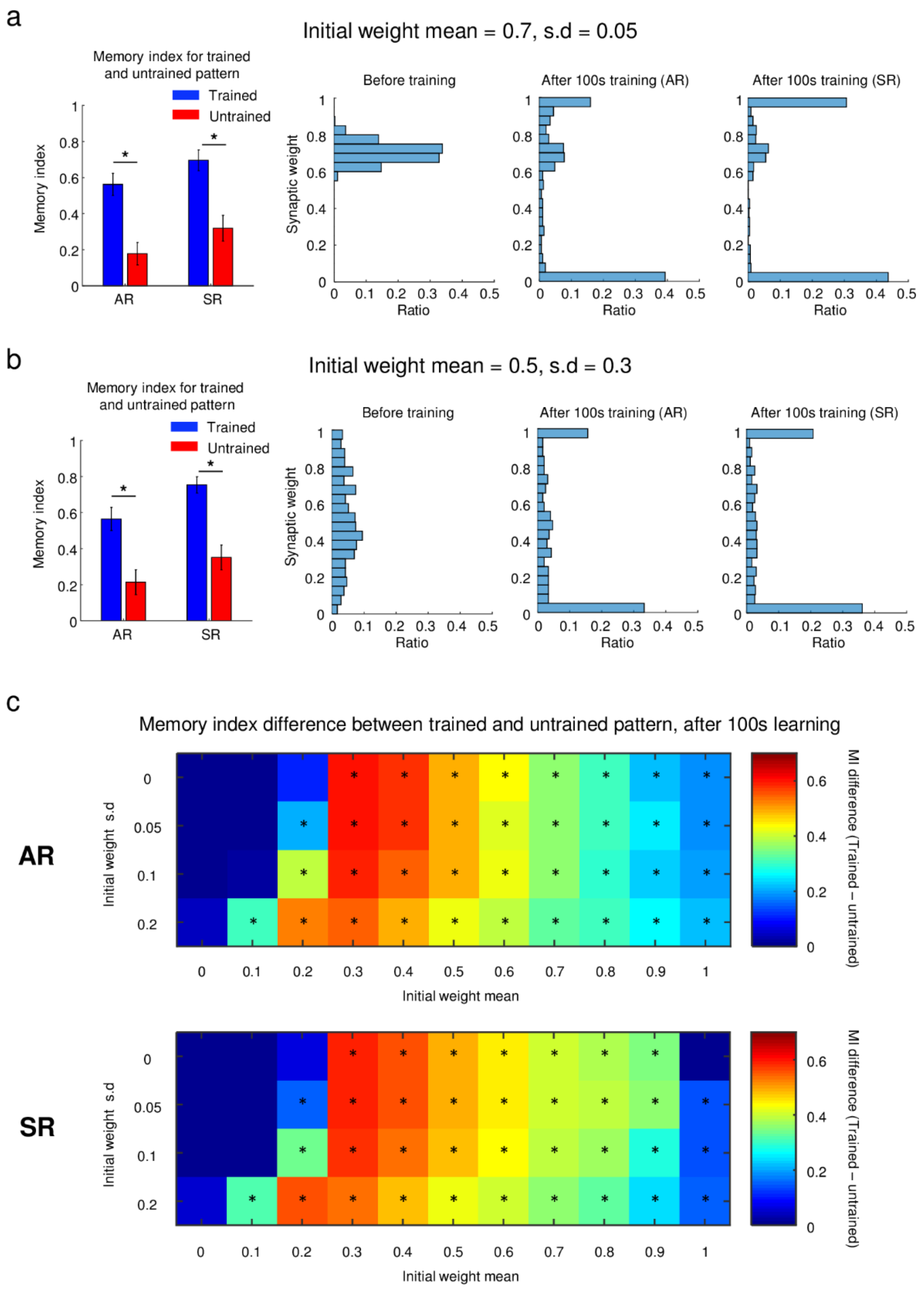

Supplementary Fig. 3. The distinction between trained and untrained patterns is robust regardless initial weights. The training was performed for 100 seconds (n=100). (a) Larger initial weights (mean=0.7, s.d=0.05) were used. (b) Larger variability of initial weights (mean=0.5, s.d=0.3) were used. (c) Memory

index difference between trained and untrained pattern, after 100s learning. Except for the conditions where no output spike was generated ($w \leq 0.1$) and all synapses were equal to 1 ($w=1$, SR), the MI of the trained pattern was significantly higher than the MI of the untrained pattern. (Mann-Whitney U-test: * $p < 10^{-16}$, $n = 100$)

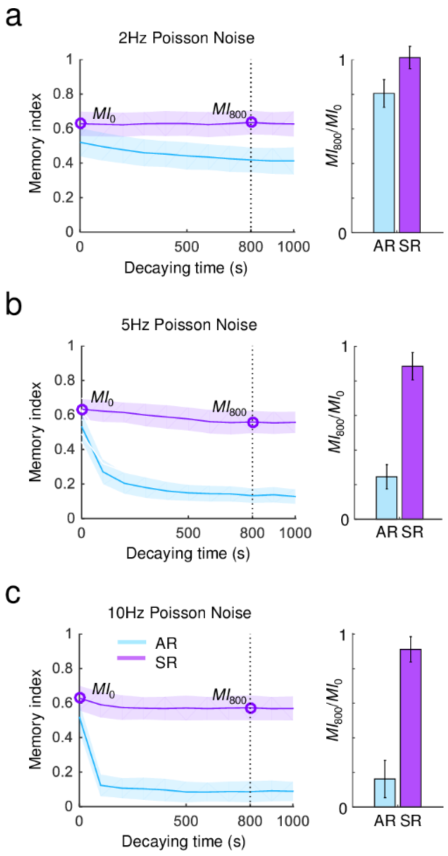

Supplementary Fig. 4. Temporal decay of memory with varied firing rates of Poisson noise. After 100 seconds of training with input patterns, Poisson spikes of (a) 2 Hz (b) 5 Hz (c) 10 Hz were introduced to networks for 1,000 seconds to erase memories created by trained patterns. In all cases, the memory index of the AR network decayed significantly while that of SR network did not show noticeable dacay (Mann-Whitney U-test: * $p < 10^{-16}$, n = 100). The shaded area and error bars represent the standard deviation.

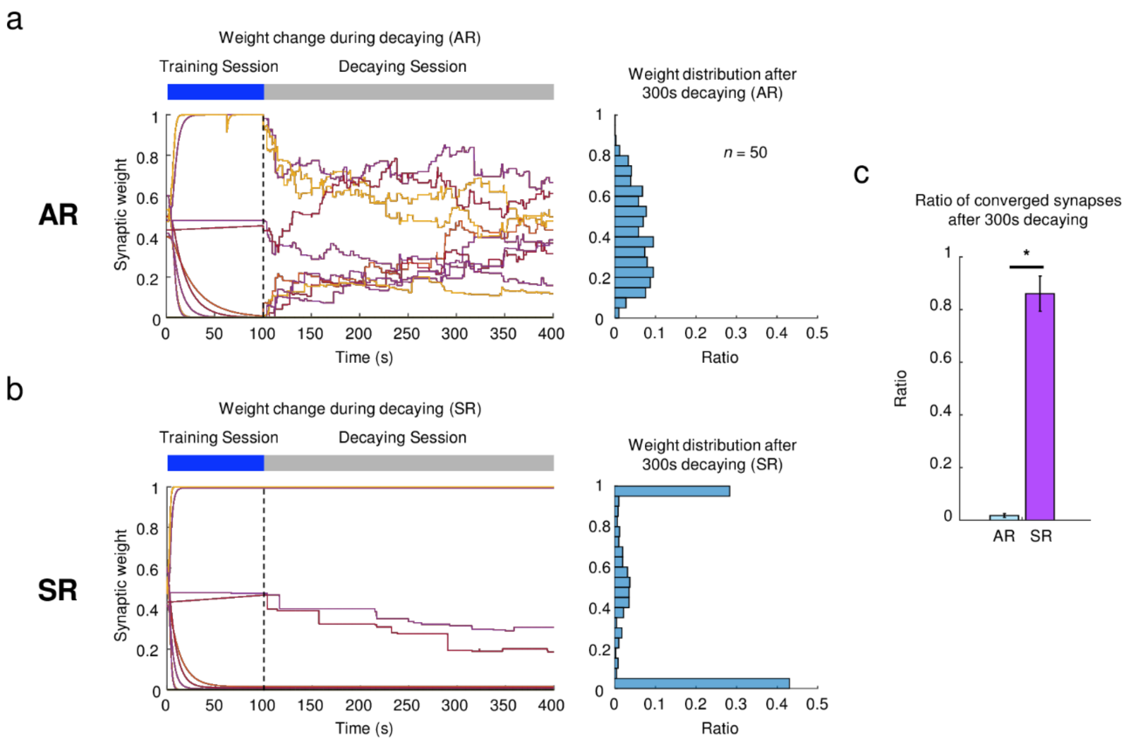

Supplementary Fig. 5. Weight change during decaying. (a) During training session, synaptic weights become bimodal as previously shown. For the AR, bimodally converged synapses after training were initialized to a median value by 10Hz noise input. (b) For the SR, bimodally converged synapses after training were maintained its value despite the presence of noise inputs. (c) Ratio of bimodally converged synapses after 300s decaying. The error bar represent the standard deviation (Mann-Whitney U-test: * $p < 10^{-16}$, n=100).

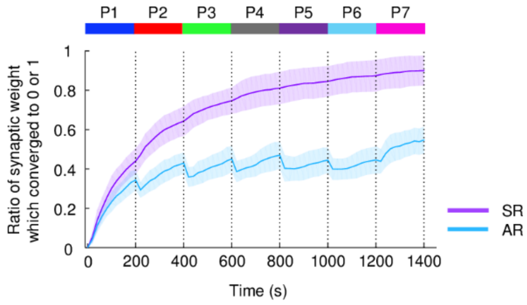

Supplementary Fig. 6. Ratio of synaptic connections that converge to either ‘0’ or ‘1’ while training the SR and AR model networks with seven consecutive patterns: In the SR model, the ratio of synapses converged to ‘0’ or ‘1’ increase continuously, as the number of appended patterns increased. In the AR model, this ratio initially increased up to ~0.4 for the training of the first pattern, but did not increases further after this.

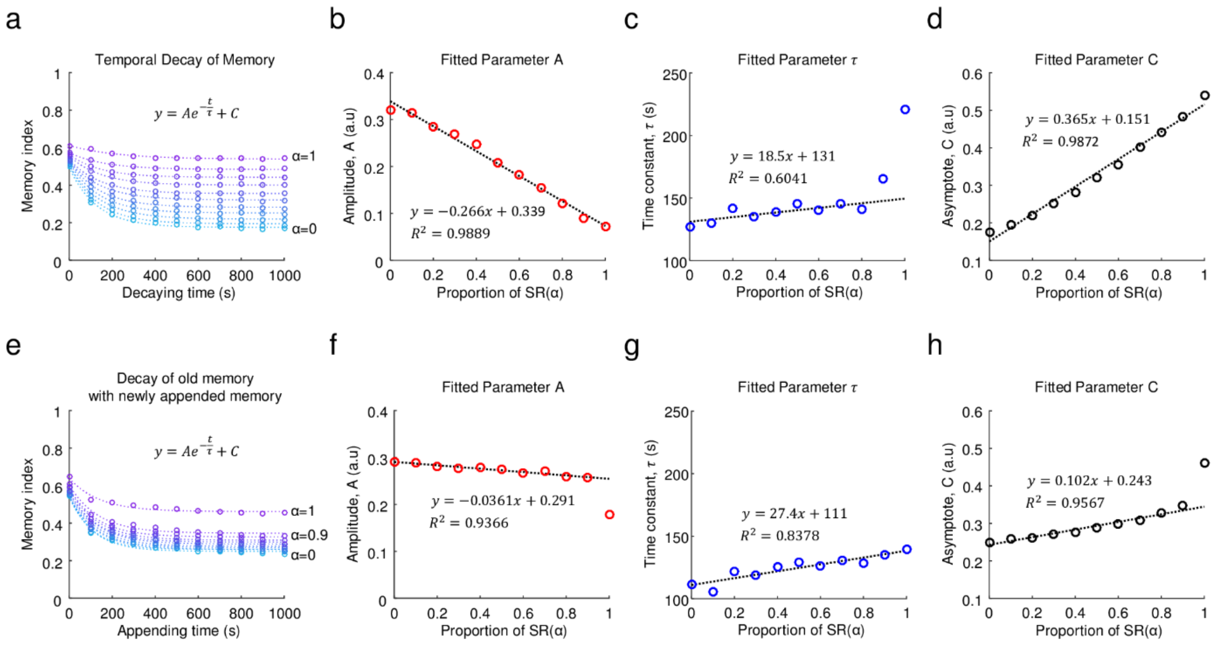

Supplementary Fig. 7. Exponential fitting of memory decay curve for various α, during the decaying test (a-d) and appending test (e-h). As α varied from 0 to 1, fitted parameters, amplitude A (b and f), time constant τ (c and g), and asymptote C (d and h) were estimated from each curve of the memory index. In (c), the data points α=0.9 and 1 were excluded from linear fitting, and in (f) and (h), data point α=1 was excluded, where a nonlinear transition of parameters was observed.

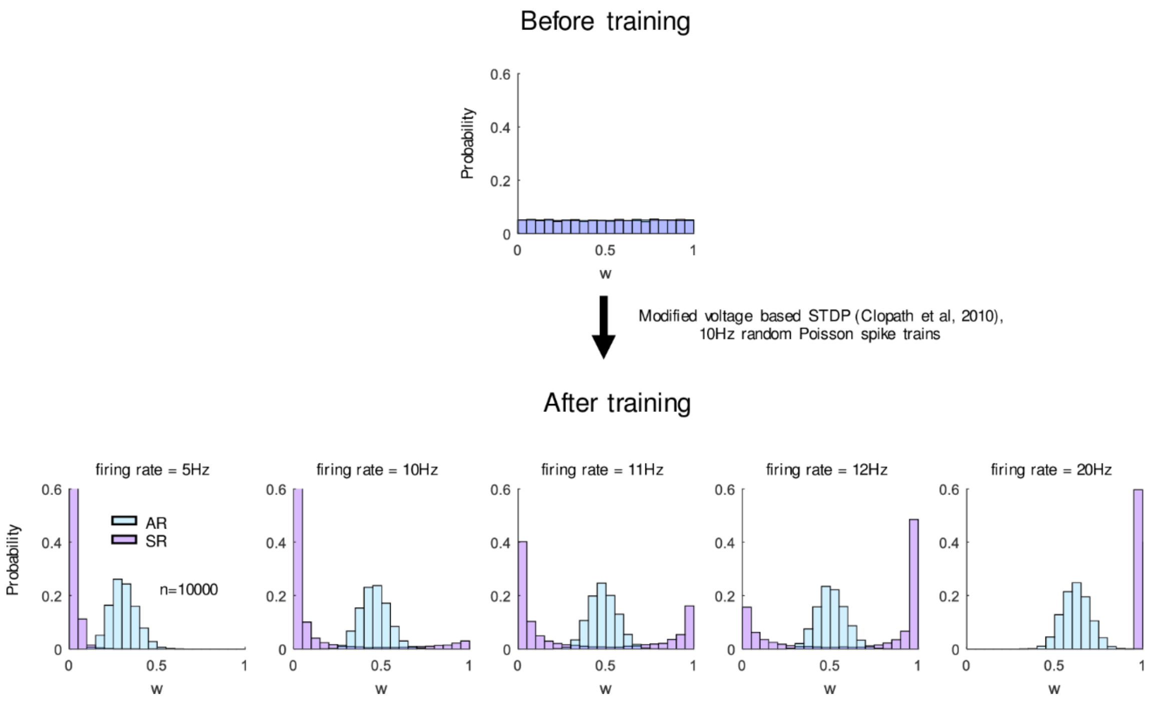

Supplementary Fig. 8. Single synapse model simulation constructed with the modified voltage-based STDP model. We performed additional simulations using a more realistic voltage-based STDP model proposed by Clopath et al. at 2010, which describes the presynaptic frequency dependence and postsynaptic voltage dependence of plasticity. The voltage-based model was originally hard bounded at ‘0’ and ‘1’ as shown in the following equation, so we modified it as SR or AR model conditions, to describe different stability profiles.

Original voltage based STDP model (Clopath et al, 2010)

$$\frac{d}{dt} w_i^- = -A_{LTD}(\bar{\bar{u}}) X_i(t)(\bar{u}_- - \theta_-)_+ \quad \text{if } w_i > w_{\min}$$

$$\frac{d}{dt} w_i^+ = A_{LTP} \bar{x}_i (u - \theta_+)_+ (\bar{u}_+ - \theta_-)_+ \quad \text{if } w_i < w_{\max}$$

Weight-dependent learning rates (AR, SR)

$$\varepsilon_{AR+}(w_{ij}) = w_{\max} - w_{ij}$$
$$\varepsilon_{AR-}(w_{ij}) = w_{ij} - w_{\min}$$

$$\varepsilon_{SR+}(w_{ij}) = \varepsilon_{SR-}(w_{ij}) = 2 \cdot \min\left(w_{\max} - w_{ij}, w_{ij} - w_{\min}\right)$$

Modified voltage based STDP model

$$\frac{d}{dt}w_i^- = -A_{LTD}(\bar{\bar{u}})X_i(t)(\bar{u}_- - \theta_-)_+ \varepsilon_-(w)$$
$$\frac{d}{dt}w_i^+ = A_{LTP}\bar{x}_i(u - \theta_+)_+(\bar{u}_+ - \theta_-)_+ \varepsilon_+(w)$$

To perform test simulations with this model, we used a single synapse model constructed with the modified voltage-based STDP model. As in the single synapse simulation above (Figure 1d–f), an input neuron was connected to an output neuron and both neurons were driven by 10 Hz random Poisson spike trains, so that spike pairs of input and output neurons for LTP and LTD were generated randomly. Then the synaptic weight was updated using the above voltage-based STDP rule.

As a result, we confirmed that the voltage-based model works in the same way as our original pair-based model, even though it has frequency dependence as in the figure above. In the voltage model with SR, synapses converged to ‘0’ or ‘1’, while AR converged to 0.5. In other words, at the single synapse level, SR was stable at ‘0’ and ‘1’, while AR was stable at 0.5, identical to the result of pair-based model. Thus, we concluded that our simulation result with pair-based model would not be significantly different from that with the voltage model, and probably with other realistic models, as long as their instability profiles can be controlled in a way similar to that in our current design.
